# Response to Jia and Wang

**DOI:** 10.1093/cid/ciaa1390

**Published:** 2020-11-10

**Authors:** Xu Liu, Qi Liu, Xueting Yao, Miao Zhang, Cheng Cui, Haiyan Li, Dongyang Liu

**Affiliations:** 1 Drug Clinical Trial Center, Peking University Third Hospital, Beijing, China; 2 Savaid Medical School, University of Chinese Academy of Sciences, Beijing, China; 3 Department of Orthopedics, Peking University Third Hospital, Beijing, China

To the Editor—Thank you for the opportunity to respond to the letter by Jia and Wang regarding our earlier publication [2].

We appreciate the comments made by Jia and Wang, especially those recognizing our novel strategy of integrating the in vitro activity and lung concentration of hydroxychloroquine (HCQ) using a physiologically based pharmacokinetic (PBPK) model to optimize dose regimens. The time between the determination of anti–severe acute respiratory syndrome coronavirus 2 (SARS-CoV-2) activity of HCQ in vitro and the recommendation of dose regimens of HCQ and chloroquine (CQ) using PBPK simulations were less than 1 week, and our clinicians almost immediately used these recommended human doses to evaluate drug efficacy and safety in coronavirus disease 2019 (COVID-19) patients in China (ChiCTR2000029899). This would be extremely difficult without PBPK models.

We agree with Jia and Wang that the “application of PBPK … must rely on rigorous pharmacokinetic mechanism and reasonable assumption.” We declared assumptions and limitations of the model, and indicated that future studies are underway to update the models [2].

The comment “the target tissue (lung) concentration of HCQ was overestimated and mismatched the in vitro activity (EC_50_)” suggests that Jia and Wang may not have carefully read or understood our approach and the assumptions presented in the paper. We described HCQ dose regimen optimization in the Methods section as follows: “in a recent clinical trial, 500 mg of chloroquine phosphate given twice daily was shown to be effective on study day 5 (R_LTEC_, day 5). This dosing regimen for chloroquine was used as the target for dose optimization for hydroxychloroquine.” Although we calculated the R_LTEC_ for each compound (CQ and HCQ), we ultimately used relative potency between the 2 compounds to facilitate HCQ’s dosing recommendations, rather than judging whether HCQ is effective or not. As compared to conventional methods that predict clinical efficacy based on in vitro and in vivo data of the same compound, our approach heavily relied on the emerging clinical antiviral effect by CQ (CQ was reported to be effective in 22 COVID-19 patients, as released on a clinical trial website and published later) [3–5]. Even for conventional methods, “mismatching” in vivo with in vitro data has been widely applied in drug development to understand the uncertainty of predicting in vivo efficacy/safety. The same concept has long been employed by industry and global regulators to predict clinical drug-drug interactions using different in vivo exposure measures for different interaction mechanisms. A recent analysis by Jansson-Löfmark et al [6] demonstrated a wide range of ratios of unbound trough concentration in plasma to in vitro potency for 164 marketed drugs across different indications. As such, we suspect that anyone can confidently claim a drug’s in vivo efficacy based on in vitro data before the drug efficacy is determined clinically (otherwise, we would either skip or significantly shorten Phase II clinical trials in today’s drug development).

We agree with Jia and Wang that “in vitro activity was significantly affected by experimental factors.” Unfortunately, our group was 1 of the first reporting half maximal effective concentration (EC_50_) of HCQ against SARS-CoV-2 [2]. Had we known other groups’ findings at the time we did our analyses, we would have considered them in our analyses: for example, by conducting sensitivity analyses or using average data.

Finally, we would like to reiterate our response to an earlier letter to the editor: “although one can employ modeling and pharmacology concepts to predict the likelihood of clinical efficacy from in vitro data, given the inherent limitations of any modeling approach and assumptions being made, in vitro efficacy can only be ultimately confirmed through clinical trials. To this end, any modeling analysis has to fit for purpose” [7].

## Notes

X. L. and Q. L. contributed equally to this work.

***Financial support.*** This work was supported by the Ministry of Science and Technology of the People’s Republic of China foundation for Severe Acute Respiratory Syndrome Coronavirus 2 Research (grant number 2020YFC0844500) and the Bill and Melinda Gates Foundation (grant number INV-015694).

***Potential conflicts of interest.*** D. L. has a patent pending on antimicrobial infection pharmaceutical composition and its applications. All other authors report no potential conflicts.
